# Quantitative isoform profiling using deep coverage long-read RNA sequencing across early endothelial differentiation

**DOI:** 10.1101/2025.05.30.656561

**Published:** 2025-06-02

**Authors:** David Wissel, Madison M. Mehlferber, Khue M. Nguyen, Vasilii Pavelko, Elizabeth Tseng, Mark D. Robinson, Gloria M. Sheynkman

**Affiliations:** 1 Department of Molecular Life Sciences, University of Zurich, Zurich, Switzerland.; 2 Department of Computer Science, ETH Zurich, Zurich, Switzerland.; 3 SIB Swiss Institute of Bioinformatics, University of Zurich, Zurich, Switzerland.; 4 Department of Molecular Physiology and Biological Physics, University of Virginia, Charlottesville, VA, USA.; 5 Department of Biochemistry and Molecular Genetics, University of Virginia, Charlottesville, VA, USA.; 6 Center for Digital Health, Berlin Institute of Health (BIH) at Charité - Universitätsmedizin, Berlin, Germany.; 7 Pacific Biosciences, Menlo Park, CA, USA.; 8 Center for Public Health Genomics, University of Virginia, Charlottesville, VA, USA.; 9 UVA Cancer Center, University of Virginia, Charlottesville, VA, USA.

**Keywords:** PacBio, Long-read RNA-seq, isoforms, quantification, endothelial cells

## Abstract

Long-read RNA-sequencing enables the profiling of full-length transcripts, but its quantification accuracy data has still not been robustly established. This is especially true for PacBio lrRNA-seq data, which were previously only available at low to moderate depth. Using a high-depth PacBio Kinnex lrRNAseq dataset, sample-matched with Illumina short-read RNA-seq, we performed rigorous benchmarking to characterize quantification accuracy between platforms on a dataset representing differentiation of induced pluripotent stem cells into primordial endothelial cells. We identified biases impacting transcript quantification, including inferential variability within Illumina data, which can bias transcript abundance estimates for genes with complex splicing, as well as length biases in Kinnex data. Overall, PacBio and Illumina quantifications were strongly concordant, supporting that PacBio Kinnex is a reliable method for transcriptome profiling and enabling downstream biological analyses.

## Introduction

In recent years, high-throughput RNA-sequencing (RNA-seq) technologies have enabled an improved characterization of the human transcriptome [1]. In addition, long-read RNA-seq (lrRNA-seq) technologies have begun offering the ability to capture full-length transcripts, which may enable more accurate discovery of isoforms, although direct comparisons with current short-read RNA-seq (srRNA-seq) methods have been understudied [2–5]. Benchmarking efforts, such as the Long-Read RNA-seq Genome annotation Assessment Consortium (LRGASP) and others have systematically evaluated the performance of lrRNA-seq data, highlighting particular methods and technologies for their high accuracy in the discovery of isoforms, among other tasks [4–11]. Beyond isoform discovery, comparative studies have explored the utility of both lrRNA-seq and srRNA-seq for transcript and gene quantification [3, 4, 11]. Previous methodological reports of this type either focused on Oxford Nanopore (ONT) lrRNA-seq data or only had access to Pacific Biosciences (PB) data at moderate to low depths, typically at a maximum of around 10M reads per sample [4, 5, 11]. Recently, the MAS-seq method, commercialized by PB as Kinnex, has enabled an increased throughput of up to 16-fold for PB lrRNA-seq data, based on cDNA concatenation [12]. Leveraging these developments, we collected a dataset representing one of the largest PB lrRNA-seq datasets, enabling technical comparisons of Illumina srRNA-seq and PB lrRNA-seq, as well as exploration of endothelial cell (EC) related transcriptome dynamics during their early development.

## Results

### Kinnex lrRNA-seq is technically robust and enables effective biological downstream analyses

**Figure 1:**
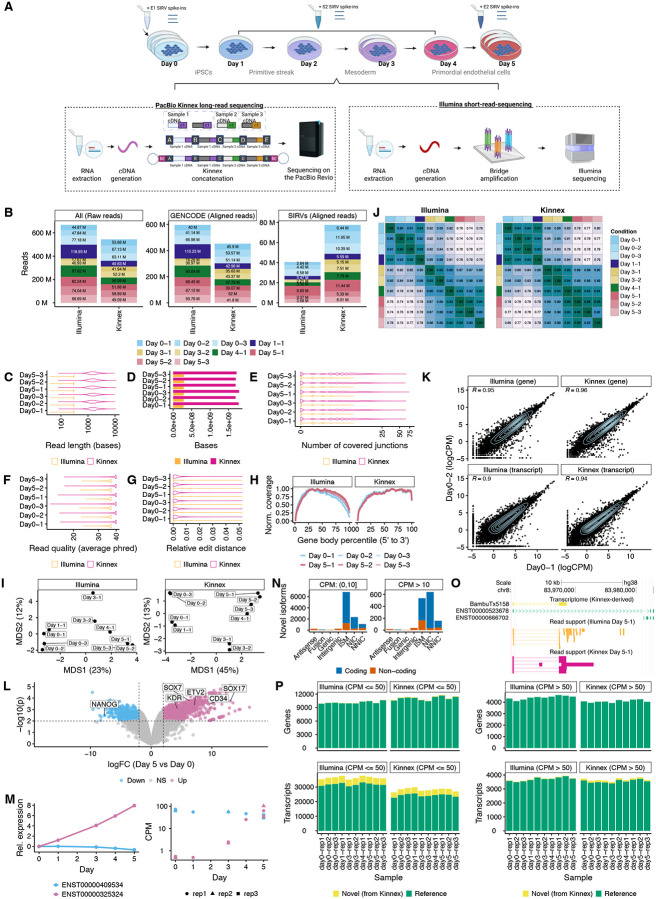
Dataset quality control, summary statistics and characterization. **A.** Experimental design to collect a matched RNA-sequencing dataset of differentiated primordial endothelial cells to track transcript dynamics over time. Kinnex preparation abbreviations (bc1 = barcode 1, bc2 = barcode 2, A, B, C and D denote Kinnex-specific primers, c1, c2 and c3 represent individual cDNA molecules) **B.** Number of raw and aligned reads per technology, stratified by their source across all samples. Day 2–1 had 0.64M aligned SIRV reads (not shown due to space). **C.** Read lengths of 1M randomly sampled reads aligning to the human genome, stratified by technology for Days 0 and 5. We highlight Days 0 and 5 due to their replication. For Illumina, the length was calculated across both ends. **D.** Number of base pairs sequenced from 1M randomly sampled reads collected for each technology (same reads as **C**). **E.** Number of covered junctions per read from 1M randomly sampled reads collected for each technology (same reads as **C**). **F.** Average base quality of 1M randomly sampled reads aligning to the human genome, stratified by technology for Days 0 and 5 (same reads as **C**). The average base quality for Illumina was determined from both ends. **G.** Relative edit distance of 1M randomly sampled reads collected for each technology (same reads as **C**).**H.** Normalized coverage of reads aligning to 10,000 randomly sampled GENCODE transcripts across gene body percentiles, stratified by technology, across all samples. **I.** Transcript-level quantification-based MDS plots highlighting sample differences across the full differentiation, stratified by technology. **J.** Spearman correlation heatmap of transcript-level quantifications highlighting sample similarities across the full differentiation for each technology. **K.** Scatter plot for Day 0–1 and Day 0–2 replicates at the gene and transcript levels following quantification, stratified by technology. **L.** Volcano plot of Differential Gene Expression between Day 0 and Day 5, highlighting genes specific to pluripotency (light blue) and primordial endothelial cells (pink). **M.** Estimated relative (left) and absolute observed (right) expression of two transcripts belonging to the *IPO11* gene across the differentiation. Relative expression estimated using a cubic spline with two degrees of freedom (see Methods). **N.** Number of novel isoforms in each category as annotated by SQANTI3, stratified by mean CPM in the two samples with the highest CPM for a particular isoform, colored by coding status as predicted by ORFanage. **O.** Browser track highlighting an example of a relatively confidently identified novel isoform that has read support in both Kinnex and Illumina data. **P.** Number of genes and transcripts detected for each technology following quantification against the Bambu-discovered transcriptome using Kinnex. Genes and transcripts were considered expressed if they had at least 1 CPM for a particular sample. Unless otherwise noted, full-depth Kinnex was quantified against the Bambu-derived Kinnex transcriptome using lr-kallisto, and full-depth Illumina was quantified against the Bambu-derived Kinnex transcriptome using Salmon (see Methods). Figure 1A was created in Biorender, Mehlferber, M. (2025) Biorender.

We collected RNA from human induced pluripotent stem cells (IPSCs) (WTC11, see Methods) that were subjected to a five-day differentiation, coaxing cells from a pluripotent state (day zero), to a primitive streak (day one), mesoderm (day three), and then a primordial EC type (early EC) [13]. For each day of differentiation, we isolated RNA with three replicates for day zero and five, two replicates for day three, and one replicate for the remaining days (one, two, and four) (Figure 1A). To assess intra-platform performance and technical validation, we included Spike-In RNA variants (SIRV) (Figure 1A, see Methods). The same samples were then subjected to parallel Kinnex lrRNA-seq and Illumina srRNA-seq (see Methods). We excluded the single day-two sample from further analysis due to quality issues (Figure S1A–J).

We first evaluated the read depth and characteristics, as well as the overall quality of the Illumina and Kinnex datasets. Both technologies reached comparable depths per sample, with Illumina averaging 65M reads per sample and Kinnex averaging 50M reads (Figure 1B). Both platforms exhibited high alignment rates to the human genome (mean ~95% for Illumina, mean ~97% for Kinnex) (Figure 1B). The Kinnex data had a higher proportion of reads aligning to the SIRV spike-ins than Illumina (Figure 1B). Importantly, the median Kinnex read length (~1.55 KB) was ~5.2 times longer than Illumina’s (299 bps, paired-end) (Figure 1C), leading to a substantially larger total number of sequenced bases per sample (Figure 1D) and higher junction coverage per read (mean of 5.6 junctions covered for PB samples compared to 0.8 for Illumina; Figure 1E). Both platforms generated high-quality reads (Figure 1F). Since base qualities are not necessarily calibrated, we calculated empirical edit distances (see Methods), showing that Kinnex and Illumina achieved mean edit distances of 0.003 and 0.006 (corresponding to empirical phred values of 25.2 and 22.2), respectively, (Figure 1G). Kinnex data exhibited a moderate 3’ bias, while Illumina did not manifest a bias at either end (Figure 1H). These quality metrics confirm that both Kinnex and Illumina datasets were technically robust.

In addition to technical quality, we confirmed that each dataset captured the expected biological progression associated with endothelial differentiation. For both Illumina and PB, MDS plots revealed tight clustering within replicates while capturing the biological differentiation along the axis of the first MDS direction (Figure 1I). Within each sequencing technology, we observed divergence of transcript expression profiles over the course of differentiation and high reproducibility of gene and transcript quantification for replicates of the same day (Spearman > 0.9) and (Figure 1J–K). The Kinnex data captured expected transitions across the differentiation, including downregulation of the pluripotency gene *NANOG* and upregulation of several EC markers, including *KDR*, *ETV2*, *CD34*, *SOX7*, and *SOX17*, by day five (Figure 1L and Supplementary Table S1) [14–18]. Exploration of Differential Transcript Usage (DTU) revealed several dominant isoform switches across the differentiation, for example within the *IPO11* gene (Figure 1M and Supplementary Table S2). These results support that both datasets reliably captured the biological structure of the model system.

Next, we evaluated how well Kinnex and Illumina detected transcripts and discovered novel isoforms during differentiation. We observed that Illumina was less accurate for isoform discovery based on the SIRV data as evidenced by poor precision and recall (Figure S3A). Some of the SIRV isoforms that were only discovered by Kinnex tended to have higher inferential variability in Illumina quantification than isoforms found by both technologies, although the pattern was not fully clear (Figure S3B). Thus, isoform discovery was performed using the Kinnex data.

Using Kinnex data from all samples (see Methods), we discovered 12,341 novel isoforms and 515 novel genes (Figure 1N and Supplementary Table S3). SQANTI annotation along with Coding DNA Sequence (CDS) prediction and quantification of our Kinnex-derived transcriptome (see Methods) revealed that while 10,935 novel isoforms (89%) were relatively lowly expressed (using the mean expression in the two most highly expressed samples) at less than 10 counts per million (CPM) and an additional 69 isoforms (0.56%) had more robust expression but belonged to categories beyond standard multi-exonic, protein-coding genes (e.g., antisense, fusion, genic, and intergenic transcripts), we found that 1,337 isoforms (10.8%) had relatively robust expression (10 CPM or higher) and belonged to more realistic SQANTI categories (ISM, NIC or NNIC) (Figure 1N) [19, 20]. In addition, 1,097 of high-confidence isoforms were predicted to contain a viable coding region (8.9% of all novel isoforms) (see Methods). BambuTx5158 represents an exemplary case, a predicted novel non-coding isoform from the ENSG00000254202 gene (found near loci for the *RP11* gene), which was moderately expressed with a CPM of 34, with clear read support from both Illumina and Kinnex (Figure 1O).

For detection, we quantified Kinnex and Illumina data against the GENCODE transcriptome, augmented with novel isoforms discovered by Kinnex (see Methods). We considered a transcript as detected for a particular day and technology if it had a minimum of one CPM in that sample. Illumina detected an average of 40,175 transcripts (see Methods) and 14,571 genes, 4,656 and 54 of which were novel (based on discovery via Kinnex), respectively (Figure 1P). Meanwhile, Kinnex had an average of 31,908 expressed transcripts and 13,155 expressed genes, 4,027 and 77 of which were novel. Although Illumina detected more lowly-expressed transcripts (<50 CPM; 36,441 vs. 28,235), detection rates were similar for higher-expressed transcripts (>50 CPM; 3,734 for Illumina vs. 3,673 for Kinnex) (Figure 1P).

### Oarfish performs best among Kinnex quantification methods when considering SIRV spike-ins and GENCODE data

To perform an in-depth characterization of transcript quantification, we first attempted to determine suitable quantification tools for Kinnex data, as long-read analysis strategies have continued to evolve [22]. We processed Kinnex data using five recently proposed methods (Oarfish, lr-kallisto, Salmon, IsoQuant, Bambu), evaluating performance primarily using the SIRV spike-ins as a ground-truth (see Methods), downsampled to the same number of reads for both Kinnex and Illumian (see Methods), and, when relevant, contrasting with Illumina results as a comparative reference (also downsampled to the same depth between platforms) [2, 3, 23–25].

**Figure 2:**
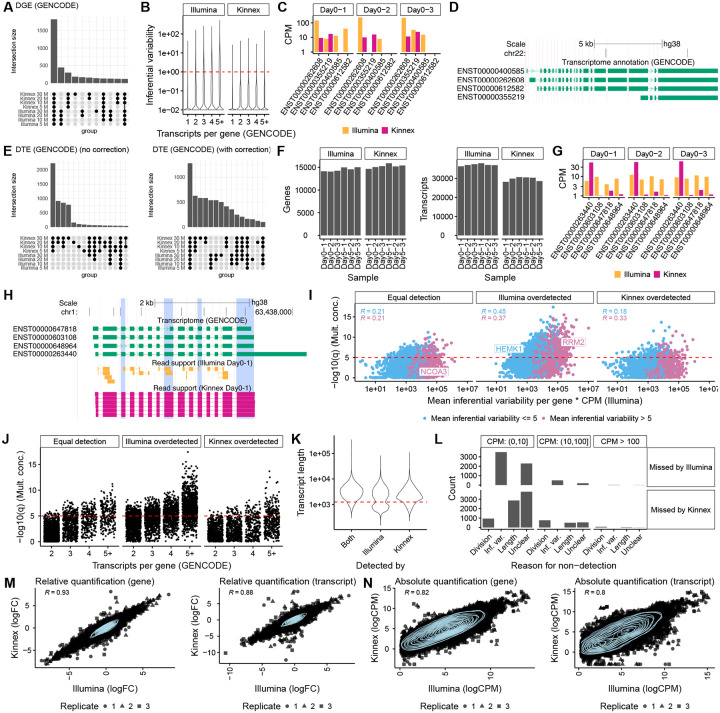
Quantification comparison between Kinnex and Illumina. **A.** Overlap of DGE calls between Day 0 and Day 5 based on Oarfish Kinnex and Salmon Illumina when downsampling GENCODE-aligned data to a fixed read depth for each replicate. **B.** Inferential variability (see Methods for GENCODE transcripts according to number of transcripts per gene, stratified by technology. **C.** Example of a gene with a ‘flip’, characteristic of transcripts with high inferential variability in Illumina, where Illumina assigns moderate CPM values to a transcript in one replicate, while for another replicate, that transcript has close to zero CPM. **D.** Transcript-model corresponding to the ‘flip’ example (**C**). **E**. Overlap of DTE calls between Day 0 and Day 5 based on Oarfish Kinnex lrRNA-seq and Salmon Illumina RNA-seq when downsampling GENCODE-aligned data to a fixed read depth for each replicate, stratified by whether Illumina counts were corrected for inferential variability or not (see Methods) [21]. **F.** Number of detected transcripts and genes for each replicate of Day 0 and Day 5 when downsampling to 30M reads, stratified by technology. **G.** Example of a ‘division’, where Illumina evenly divides count mass between transcripts with high sequence similarity, whereas Kinnex assigns most count mass to a singular transcript. **H.** Transcript-model corresponding to the ‘division’ example (**G**), with read support for both Illumina and Kinnex, highlighting that both technologies primarily show read support for the ENST00000263440 transcript. **I.** Mean inferential variability per gene times CPM in Illumina by the −log10(q) value of testing for multinomial concordance between Illumina and Kinnex, stratified by whether the two platforms detected an equal number of transcripts (equal detection) for the gene in question, or Illumina detected more transcripts (Illumina overdetected) or Kinnex detected more transcripts (Kinnex overdetected). **J.** Number of transcripts per gene by the −log10(q) value of testing for multinomial concordance between Illumina and Kinnex, stratified by whether the two platforms detected an equal number of transcripts (equal detection) for the gene in question, or Illumina detected more transcripts (Illumina overdetected) or Kinnex detected more transcripts (Kinnex overdetected). **K.** Distribution of transcript length for transcripts that are detected by both technologies and transcripts detected only by Kinnex or Illumina, highlighting that Kinnex shows a clear length bias for transcripts shorter than 1,250 nts. **L.** Number of transcripts missed by each technology and possible reasons for their non-detection, stratified by average CPM in the technology in which they were detected. **M.** Relative quantification (log-fold change) concordance between Kinnex and Illumina on the gene and transcript level, per replicate, when filtered for transcripts and genes that show low inferential variability in Illumina and are detected by both platforms. **N.** Absolute quantification (log CPM) concordance between Kinnex and Illumina on the gene and transcript level, per replicate, when filtered for transcripts and genes that show low inferential variability in Illumina and are detected by both platforms. Unless otherwise noted, Kinnex was downsampled to 30M reads and quantified against GENCODE V45 using Oarfish. Illumina was also downsampled to 30M reads per sample and quantified against the same transcriptome using Salmon (see Methods). R denotes Pearson correlation.

In evaluations of computational resource usage, Oarfish and lr-kallisto stood out with fast runtimes and low memory usage across read depths (Figure S2A). Quantification performance on the SIRV data was assessed across three metrics: (1) Differential Transcript Expression (DTE) accuracy, (2) relative quantification accuracy, and (3) absolute quantification accuracy. For DTE, accuracy was defined as the ability to correctly identify differentially expressed transcripts between two conditions. Given this criteria, we found that Isoquant and lr-kallisto performed competitively with Illumina on the SIRV data, achieving comparable True Positive Rate (TPR) and lower False Discovery Rate (FDR) (Figure S2B). Although Bambu, Oarfish, and Salmon appeared to perform worse overall (Figure S2B), closer inspection revealed that the performance drop for Oarfish, and partly, Bambu, was mostly driven by a single outlier transcript (SIRV303), characterized as having high sequence similarity to other SIRVs in the mixture (Figure S2C), rather than widespread inaccuracies, suggesting possible algorithmic vulnerabilities. Our findings for DTE accuracy were mirrored for the relative quantification accuracy, defined as the agreement between known versus measured fold changes. Isoquant and lr-kallisto again performed well, achieving Pearson correlation values similar to Illumina, whereas Bambu and Oarfish were hampered by the SIRV303 outlier, and Salmon exhibited poor performance overall (Figure S2D). All Kinnex-based quantification methods exhibited a slight length bias for SIRV transcripts shorter than ~1,250 nucleotides, suggesting that this bias may be inherent in the Kinnex library (Figure S2D). Finally, for absolute quantification on the SIRVs, defined as the agreement between known and measured log CPM values, all Kinnex-based methods demonstrated moderate concordance with Pearson correlations around ~0.70, compared to higher correlations achieved by Illumina (~0.88) (Figure 2E).

Recognizing that SIRV spike-ins do not fully capture transcriptomic complexity, we next assessed quantification performance between platforms, focusing on endogenous human GENCODE transcripts. We evaluated performance based on the relative quantification efficiency and the number of significant DTE calls reproducibly supported across quantification methods and platforms (see Methods). Quantification efficiency was calculated as the proportion of quantified counts to raw input reads at a subsampled read depth (see Methods). To account for Illumina’s known challenges in resolving of highly similar transcripts, i.e., inferential variability, we evaluated Illumina results both with and without a scaling-based inferential variability correction [21, 26–28]. Across both metrics, Oarfish outperformed all other methods, achieving efficiencies close to one, with higher numbers of reproducible DTE calls than other methods, including Illumina with correction (Figure S2F–G). Based on these results, as well as its favorable performance in terms of computational resource needs as well as quantification accuracy on the SIRV spike-ins (besides the SIRV303 outlier), we selected Oarfish for all downstream Kinnex-based analyses. We used Salmon for Illumina-based analyses (see Methods).

### Kinnex mitigates inferential variability-related quantification issues encountered by Illumina data, but suffers from length biases

To establish a baseline for platform comparison, we first evaluated Differential Gene Expression (DGE), which remains well-established and simpler than transcript-level analyses. We observed that differential gene expression calls (see Methods) were highly concordant between Illumina and Kinnex across downsampled read depths (Figure 2A), indicating similar performance for gene-level analyses.

We next assessed the more challenging task of transcript-level quantification. Consistent with previous findings [21, 29], Illumina exhibited substantially higher inferential variability compared to Kinnex, particularly for genes with more transcripts (Figure 2B). This variability was reflected by greater replicate-to-replicate fluctuations of estimated transcript abundances from the short-reads when attempting to resolve highly similar transcripts (Figure 2C) (see Methods) [21]. A manifestation of this is the occurrence of ‘transcript flips’, where replicate quantifications for Illumina alternate between moderate-to-high or near-zero values for a given transcript, whereas Kinnex demonstrated consistent quantification for the same transcripts. An example is the *CECR2* gene, in which all four transcripts are highly similar in sequence, differing only by short exons and alternative acceptor sites (Figure 2C–D). Illumina quantifications frequently exhibited transcript flips across replicates, while Kinnex data did not suffer from this problem and tended to assign expression to one or multiple dominant transcripts and near-zero expression to the others, as evidenced by its overall notably lower inferential variability (Figure 2B–D).

Instability caused by inferential variability in Illumina quantifications impacted downstream analyses. When computing DTE calls, Illumina initially yielded far fewer significant events than Kinnex-based Oarfish quantifications (Figure 2E), concordant with previous research [21]. The low number of calls was attributed to Illumina’s challenges in reliably quantifying transcript abundances for genes with complex splicing structures, where high inferential variability dilutes statistical power. To address this, we applied an inferential variability correction (see Methods), an ‘adjustment’ that down-weights transcripts with highly uncertain quantification [21]. After adjustment, the concordance between Illumina and Kinnex DTE calls improved substantially, both in the number of events captured and q-value distributions (Figure 2E, Figure S3C). Because of this, all transcript-level Illumina analyses employed this adjustment, including those previously shown in Figure 1 [21] (exception: figures that highlight quantification challenges, such as Figure 2C, G, and Figure S3F–H).

To further assess platform performance, we compared the number of detected genes and transcripts for each replicate, downsampled to 30M reads (see Methods). Kinnex detected a slightly higher number of genes on average (15,253 vs. 14,506 for Illumina), but notably fewer transcripts (29,721 vs. 37,163; Figure 2F). Upon closer investigation, however, we noticed examples in which the additional transcripts detected by Illumina may have been spurious. For the *ALG6* gene, Kinnex identified a single dominant transcript (ENST00000263440), assigning minimal expression to all other transcripts, whereas Illumina divided expression more evenly across all four transcripts (Figure 2G), despite little to no unique read support for any single transcript (Figure 2H). This suggests that higher transcript counts from Illumina could sometimes be misleading, reflecting quantification noise from short-read ambiguity rather than true biological complexity. We quantified the level of differences in gene proportions between Illumina and Kinnex by evaluating multinomial concordance between the two platforms (see Methods). Overall, the gene-level q-value for multinomial concordance only showed a moderate positive Pearson correlation with the mean inferential variability for that gene (once scaled by CPM, since the inferential variability measure we used attempts to be independent of the mean, see Methods [30]) (Figure 2I). Correlation was notably higher for genes where Illumina detected a higher number of transcripts (Illumina overdetected), supporting our heuristic ‘transcript division’ example (Figure 2G). Nevertheless, we found several example genes that exhibited almost arbitrary combinations of high or low inferential variability combined with high or low multinomial concordance gene-level q-value, suggesting that inferential variability and transcript ‘division’ may be separate phenomena (Figure S3F–H). Similar to inferential variability, the gene-level q-value associated with multinomial concordance tended to increase with the number of transcripts per gene, especially for genes for which Illumina detected more transcripts than Kinnex (Figure 2J). Kinnex also exhibited certain biases. As observed with SIRV spike-ins, Kinnex exhibited a tendency for reduced detection rates of short transcripts less than 1,250 nucleotides (Figure 2K).

Together, our analyses suggest at least two reasons why quantification differed between platforms. First, Illumina’s short-read limitations led to unreliable quantifications for complex genes, manifested either as transcript flips across replicates or transcript division of expression among multiple similar transcripts. Second, Kinnex exhibited reduced detection efficiency for shorter transcripts, as seen in both spike-in controls and endogenous genes. Despite these biases, transcripts detected by only one platform tended to be lower expressed, especially when no clear technical explanation was evident (Figure 2L).

Having established the sources of platform-specific transcript detection differences, we then evaluated how well Illumina and Kinnex quantifications agreed for transcripts reliably detected by both platforms (see Methods), leaving 8,797 transcripts and 12,197 genes for comparison, respectively. We note that we ended up with a lower number of transcripts than genes, since evaluating concordance of transcript-level quantification required an additional inferential variability filter, which was not necessary for gene-level quantification (see Methods). We observed that relative quantification (log-fold change estimates) showed very high concordance, with Pearson correlations exceeding 0.9 at the gene level and approaching 0.9 at the transcript level (Figure 2M). These results indicate that Kinnex quantifications closely matched Illumina for transcripts that could be considered as reliably quantified by both platforms. Absolute quantification (log CPM values) showed moderately lower concordance, but still exceeded correlation values of 0.8 at the gene level and 0.79 at the transcript level (Figure 2N). Both relative and absolute quantification again showed a small to moderate length bias for the Kinnex data, with shorter transcripts showing a somewhat lower correlation (Figure S3D–E).

## Discussion

Overall, our results supported strong quantification performance for Kinnex. Similarly to other long-read technologies [21], Kinnex largely seemed to avoid problems of inferential variability and detected more DTE events than Illumina, even with correction. Even when applying a correction for inferential variability, Illumina suffered from issues such as transcript flips and divisions of expression, which may confound downstream analyses such as DTE. For transcripts reliably quantified across both platforms, Kinnex and Illumina showed high concordance, supporting Kinnex as an effective alternative for transcript-level studies. Nonetheless, Kinnex exhibited some limitations, particularly showing a bias against shorter transcripts and exhibiting a slightly lower performance in absolute compared to relative quantification. In addition, Kinnex and similar lrRNA-seq technologies are typically significantly more expensive than Illumina for the same sequencing depth.

Our study has some limitations. First, our dataset and analyses are a comparison between Illumina srRNA-seq and Kinnex lrRNA-seq, preventing direct comparisons to ONT data. In addition, our work does not constitute a fully exhaustive benchmark of currently existing quantification methods for Kinnex. Future work should expand these comparisons by generating datasets that encompass srRNA-seq, Kinnex, and ONT data at comparable depth, and including a broader array of lrRNA-seq quantification methods, enabling exhaustive quantification evaluations for the field.

## Methods

### Dataset generation

#### Stem cell culture

Undifferentiated human-induced pluripotent stem cells (hiPSCs, WTC11, NIGMS Repository Number GM25256) were obtained through Coriell via a Materials Transfer Agreement with the University of Virginia. WTC11 cells were thawed from Cryostor (Stem Cell Technologies) and cultured using mTESR Plus Media (Stem Cell Technologies) on Matrigel (Corning) coated plates with media changes following manufacturer recommendations. Cells were cultured to maintain undifferentiated cell populations and passaged when confluent using ReLSR (Stem Cell Technologies) and replated at desired densities per manufacturer guidelines on Matrigel-coated dishes.

#### Stem cell (WTC11) derived primordial endothelial cells

WTC11 cells were seeded with mTESR Plus Media (Stem Cell Technologies) onto 6-well dishes or 10cm Matrigel-coated plates for primordial endothelial cell differentiation as described previously [13]. Twenty-four hours after plating, on Day 0 media was aspirated and replaced with Diff APEL 2 (Stem Cell Technologies) differentiation media containing 5 μm GSK3i (CHIR99021, Reprocell). On Day 1 media was aspirated and replaced with differentiation media containing 50ng/mL essential fibroblast growth factor (bFGF, R and D Systems). On Days 2, 3, and 4 media was aspirated and replaced with differentiation media containing 25ng/mL of bone morphogenetic protein 4 (BMP4, R&D Systems) and 50 ng/mL of vascular endothelial growth factor VEGF (Fisher Scientific). Cells were collected from Days 0–5 of the differentiation protocol using Accutase (ThermoFisher) per manufacturer guidelines and pelleted. Three biological replicates collected on Day 0, with Day 0–1 and Day 0–2 collected from a 10 cm dish and Day 0–3 from a 6-well dish. Three biological replicates were collected as described above for Day 5, with Day 5–1 and Day 5–2 derived from a 10 cm dish and Day 5–3 from a 6-well plate. Two biological replicates were obtained from Day 3 from 10 cm dishes. Days 1, 2, and 4 were all collected from 10 cm dishes. For each sample, RNA was extracted at approximately 1 million cells using an RNeasy (Qiagen) kit, with quality assessment via Bioanalyzer. Spike-In RNA Variants (SIRVs, Lexogen) were reconstituted per manufacture guidelines and added to samples with Day 0 biological replicates receiving E1 SIRVs, Day 5 E2, and all other Days receiving S4. Master mixes of RNA and SIRVs were split into 4 tubes for parallel long-read PB Revio sequencing and short-read sequencing on the NovaSeq at 150bp.

#### qPCR gene marker analysis

Transcriptomes were collected from cultured WTC11 cells at every day of the differentiation protocol using the RNeasyPlus Micro Kit (QIAGEN). Resulting cDNA libraries were assessed via qPCR using published PCR primers (Integrated DNA Technologies, based on [13, 31]) and miScript SYBR Green PCR Kit (QIAGEN) to validate gene expression patterns associated with the intermediate states.

#### Short-read RNA-sequencing

Aliquots of total RNA samples created from the master mix preparation as described above, were used as input for sequencing to Kapa RNA HyperProKits for short-read RNA sequencing library preparation. The resulting libraries were sequenced on the NovaSeq at 150bp.

#### Kinnex long-read RNA sequencing library preparation and long-read RNA sequencing run

Aliquots of total RNA samples created from the master mix preparation as described above were used as input for PB Kinnex. From the RNA, cDNA was synthesized using the Iso-Seq Express Kit (PB). Approximately 300ng of cDNA from each sample was barcoded and equally pooled. All replicates in one WTC11 sample were made into one Kinnex full-length library and sequenced on one SMRTCell (i.e., WTC11 Day 0 and WTC11 Day 5 replicates were sequenced in one run).

### Data Analysis

#### Preprocessing

To prepare raw sequencing files for alignment, we performed different preprocessing steps per technology.

##### Illumina.

We ran Trim Galore 0.6.2 in paired-end mode with a minimum length of 20 and a minimum quality of 20 using the --phred33 option.

##### Kinnex.

Kinnex data were preprocessed on-instrument. Basecalling was performed using basecaller version 5.0 and otherwise standard parameters. CCS reads were generated using ccs 7.0.0, followed by skera demultiplexing of Kinnex S-reads using skera 1.0.99, lima 2.8.99, and Isoseq refine 3.99.99, at which point reads were ready for alignment.

#### Separating FASTQ into spike-in and non-spike-in data

As an initial step, both for QC and since spike-ins may require different alignment options, we separated all FASTQ files into reads aligning to either one of the artificial spike-in genomes or to the human genome.

##### Alignment for Illumina-generated RNA-sequencing files

Data was aligned to a concatenation of the human genome, the full SIRV genome, and the ERCC genome using STAR 2.7.11b [32]. Afterward, BAM files were separated by whether they aligned to the human or one of the artificial genomes and converted to FASTQ using samtools 2.19.2 [33].

##### Alignment for Kinnex-generated RNA-sequencing files

Data was aligned to a concatenation of the human genome, the full SIRV genome, and the ERCC genome using minimap2 2.28–0 with the splice:hq -uf options [34]. Afterward, BAM files were separated by whether they aligned to the human or one of the artificial genomes and converted to FASTQ using samtools 2.19.2.

#### Genome alignment (Kinnex)

Genome alignment was performed using minimap2 2.28–0 with the splice:hq -uf options and provided annotated splice sites using --junc-bed. For the alignment of SIRV reads, we also applied the --splice-flank option.

#### Transcriptome alignment (Kinnex)

Transcriptome alignment was performed using minimap2 2.28–0 with the map-hifi --eqx options, allowing for a maximum of 100 secondary alignments by using the -N 100 option.

#### Genome alignment (Illumina)

Genome alignment was performed using STAR 2.7.11b using the --outSAMstrandField intronMotif argument and otherwise default parameters. Indices for STAR were generated using the --sjdbGTFfile option to which appropriate transcriptome GTF files were passed. The --sjdbOverhang option was set to 150.

#### Transcriptome alignment (Illumina)

Transcriptome alignment was performed using STAR 2.7.11b using default parameters. Indices for STAR were generated using default parameters.

#### References

We used the GRCh38 genome (GCA 000001405.15_GRCh38_no_alt_analysis_set.fna.gz) as the human reference genome and the primary assembly of GENCODE V45 as the human reference transcriptome [35]. For the spike-ins, we used the genome and transcriptome corresponding to SIRV set four, including short SIRVs, long SIRVs, and ERCCs.

#### Quantification (Kinnex)

##### Isoquant.

Isoquant 3.6.1 was used to quantify genome alignments with precomputed DB files, never requiring a polyA tail, data type pacbio_ccs, and otherwise default parameters [3].

##### lr-kallisto.

kallisto 0.51.0 was used in conjunction with bustools 0.44.1 to quantify FASTQ files using an index k-mer setting of 63, a bus threshold of 0.8, and PacBio as the platform (-P PacBio) [24].

##### Oarfish.

Oarfish 0.6.2 was run to quantify transcriptome alignments with model coverage applied, no filters, and otherwise default parameters [25].

##### Bambu.

Bambu 3.8.0 was run to quantify genome alignments with default parameters [2].

##### Salmon.

Salmon 1.10.3 was run to quantify transcriptome alignments with the --ont flag and otherwise default parameters.

#### Quantification (Illumina)

For Illumina data, we used Salmon 1.10.3 to quantify FASTQ files using an index k-mer setting of 31, a fragment length prior mean of 250, a fragment length standard deviation of 25, the --validateMappings --gcBias --seqBias flags, and otherwise default parameters [23].

#### Inferential variability correction

For Illumina, we corrected for inferential variability using the catchSalmon function in edgeR 4.4.0. Kinnex quantifications were not corrected for inferential variability [21, 36].

#### Gene-level quantificationss

Gene-level quantifications were generated by summing all transcript-level quantifications for all transcripts belonging to a gene, according to a particular annotation.

#### Quality control

##### Read count numbers.

Read count numbers were calculated using samtools 2.19.2. For aligned data, we counted primary alignments after separation into SIRV and GENCODE data.

##### Read length and number of bases.

Read length was calculated using bioawk 1.0 as the number of nucleotides in each read for Kinnex and the sum of nucleotides in each paired-end read for Illumina. Number of bases was calculated as the sum of read lengths for each technology.

##### Number of covered junctions.

Covered junctions were calculated as the number of introns of length 20 or longer in the cigar string of the genome alignments of each technology using pysam 0.23.0.

##### Read quality and relative edit distance.

Read quality was calculated as the average phred value of all bases of that read for Kinnex or the average phred of all bases in each paired-end read for Illumina using bioawk 1.0.

Relative edit distance was heuristically calculated based on the BAM file as the edit distance from the nM tag divided by the total number of aligned bases for Kinnex and the sum of edit distances divided by the sum of the total number of aligned bases in each paired-end read for Illumina using pysam 0.23.0.

##### Gene body coverage.

Gene body coverage was calculated on genome-aligned BAM files using 10,000 randomly subsampled GENCODE transcripts using RSeQC 5.0.4 [37].

###### Fragment size.

For Illumina data, fragment size was calculated on transcriptome-aligned BAM files by using the TLEN attribute of the BAM file using pysam 0.23.0.

#### Transcript and gene filtering

Depending on the analysis, we performed different types of transcript or gene filtering. SIRV transcripts were never filtered.

##### Replicability (Figure 1 J–K).

Only transcripts or genes having at least one CPM in at least three samples in both platforms were kept.

##### QC detection (Figure 1P and Figure 2F).

A transcript or gene was considered as detected if it had at least one CPM in the sample in question.

##### Differential analyses.

For all differential analyses, we used the filterByExpr function of edgeR 4.4.0 with default parameters.

##### Technology detection (Figure 2I–L).

Transcripts were considered as detected if they had at least one CPM in all three replicates of day zero.

##### Relative quantification concordance (Figure 2M).

Transcripts were kept if they had inferential variability less than five for the Illumina data, and they had at least one CPM in all three replicates of day zero in both technologies. Requirements for genes were identical except that no inferential variability filter was applied.

##### Absolute quantification concordance (Figure 2N).

Transcripts were kept if they had inferential variability less than one for the Illumina data, and they had at least one CPM in all three replicates of day zero in both technologies. Requirements for genes were identical except that no inferential variability filter was applied.

#### Isoform discovery (GENCODE)

Bambu 3.8.0 was run to perform isoform discovery using Kinnex genome alignments, selecting the Bambu-suggested NDR and keeping ISM novel isoforms (remove.subsetTx = FALSE).

#### Isoform discovery (SIRVs)

Isoform discovery on the SIRVs was ran on SIRV genome alignments, downsampled to 2.5 M reads for both platforms. Genome alignments were generated without providing the location of existing SIRV splice junctions to the aligner.

Discovery for Illumina was performed using stringtie 2.2.3 with default parameters and without a reference transcriptome. Discovery for Kinnex was performed using Bambu 3.8.0 in de-novo mode, with an NDR of 1.0 and keeping ISM novel isoforms (remove.subsetTx = FALSE) [38].

Performance was calculated using gffcompare 0.12.6 by comparing the predicted transcriptomes of both technologies to the ground truth transcriptome of the short SIRVs [39]. Performance metrics were reported at the intron chain level.

#### Annotation of novel isoforms (GENCODE)

Novel isoforms were annotated using SQANTI3 5.1.2. For determining whether a particular novel transcript was coding or non-coding, we ran ORFanage 1.1.0 in BEST mode, using GENCODE V45 CDS as a reference, and determined a novel isoform as coding if it was assigned a CDS by ORFanage [19, 20].

#### Inferential variability

We used fishpond 2.12.0 for all analyses regarding inferential variability. In particular, we used computeInfRV to calculate inferential variability as the mean inferential relative variance of each transcript across samples [30].

#### Quantification of transcript mass division between Illumina and Kinnex

To quantify differences in the division of transcript mass for a particular gene between Illumina and Kinnex, we performed a test for multinomial concordance between Illumina and Kinnex. In particular,DTU was performed with edgeR’s glmQLFit and diffSpliceDGE across all replicates of day zero of both Illumina and Kinnex, accounting for both technology and replicate. We tested significance on the technology coefficient. Transcript-level q-values were either used directly or aggregated to the gene level using Simes’ adjustment.

#### Definition of categories leading to lack of detection (Figure 2L)

##### Transcripts missed by Kinnex.

1. **Length**: Transcripts that were shorter than 1,250 nucleotides
2. **Division**: Transcripts longer than 1,250 nucleotides but having a −log10(q) of multinomial concordance between Illumina and Kinnex greater than five
3. **Unclear**: All other transcripts not detected by Kinnex but detected by Illumina

##### Transcripts missed by Illumina.

1. **Inf. var.**: Transcripts with a mean inferential variability value greater than five
2. **Unclear**: All other transcripts not detected by Illumina but detected by Kinnex

#### Downsampling

For optimal comparability in some analyses, we downsampled read FASTQ or alignment BAM files without replacement.

SIRV files were downsampled to 0.25 million, 0.5 million, 1 million, and 2.5 million, while GENCODE files were downsampled to 5, 10, 20, and 30 million, respectively. For the SIRV downsamplings, we performed five downsamplings per target depth with different random seeds, while for GENCODE only one downsampling was performed.

BAM files for genome and transcriptome alignments were downsampled using a script leveraging Rsam-tools 2.18.0 that kept all alignments corresponding to a source read. FASTQ files were downsampled using seqtk 1.4, ensuring contiguity for paired-end Illumina reads.

#### Differential analyses (GENCODE)

DGE was performed between all replicates of Day 0 and Day 5, using edgeR’s glmQLFit, accounting for both day and replicate. We tested significance on the day coefficient and called genes as significant that had a q-value less than 0.01.

DTE was performed between all replicates of Day 0 and Day 5, using edgeR’s glmQLFit, accounting for both day and replicate. We tested significance on the day coefficient and called transcripts as significant that had a q-value less than 0.01.

DTU was performed across all replicates across the timecourse (excepting Day2-1) using a cubic spline with edgeR’s glmQLFit and diffSpliceDGE, accounting for both day and replicate. We tested significance on all spline coefficients jointly and called genes as significant that had a q-value less than 0.01, based on Simes adjustment.

#### Differential analyses (SIRVs)

We performed DTE on the SIRV datasets between all replicates of Day 0 and Day 5, corresponding to SIRV mixes E1 and E2, respectively. In particular, we used the true log-fold changes between SIRV mixes E1 and E2 as the ground-truth, yielding either 0 (no change between mixes), −1 (decrease from E1 to E2), or 1 (increase from E1 to E2).

We then performed DTE using edgeR’s glmQLFit, with a design matrix accounting for both the mix and the replicate, testing the significance of the mix coefficient. Predictions were extracted using limma’s decideTests, using an FDR cut-off of 1% and no log-fold change cutoff [40]. The FDR was calculated as the percentage of SIRVs found as −1 or 1 that were either 0 or the opposing sign in the ground-truth (i.e., false discoveries) over all discoveries (i.e., all SIRVs that had a non-zero value assigned). The TPR was calculated as the percentage of SIRVs that had a ground-truth non-zero value, were deemed significant, and had the same sign as the ground-truth over all ground-truth non-zero values.

All quantifications were downsampled to a depth of one million reads for fairness. FDR and TPR values correspond to mean values calculated across all five downsamplings.

#### Relative quantification efficiency

Relative quantification efficiency was assessed for each quantification method by calculating the average proportion of quantified counts to raw input reads at several downsampled depths for the GENCODE data (5M, 10M, 20M, 30M).

#### Definition of significant DTE calls (Figure S2G)

We considered any call that had an FDR less than 0.01 and was called by at least one other quantification method at the same depth as significant.

#### Computational requirements

Computational requirements were measured using the Snakemake benchmark functionality and are reported over each of the three replicates for days zero and five. Since computational requirement variability was minimal for reruns, we do not report variability within replicate and day. All methods were run with access to 12 cores.

#### Reproducibility

All bioinformatics experiments were performed on an Ubuntu 22.04.5 LTS server with AMD EPYC 7742 CPUs. Experiments were run with Snakemake 8.2.3 within rule-appropriate mamba environments within Singularity containers [41]. All experiments are reproducible up to numerical stability via standard Snakemake commands documented on our Github (see Data Availability).

All analysis was either performed in respective command line tools or in R 4.4.3 [42]. Figures were generated using ggplot2 3.5.2 and ggpubfigs 1.1.0 [43, 44].

## Supplementary Material

1

## Data Availability

For access to the raw sequencing files for both Illumina and Kinnex, see University of Virginia (UVA), Integrated Translational Health Research Institute of Virginia (iTHRIV). Researchers who wish to gain access to the source data can contact the authors to initiate a data request. Before sharing, both groups will seek approval from the NIGMS repository by completing the Statement of Research Intent forms. Once approved, a contract can be initiated between institutions for the transfer and reuse of the source data in this study. Count tables, separated into spike-in and non-spike-in data for the GENCODE reference transcriptome, and the GENCODE reference transcriptome augmented with Kinnex lrRNA-seq-derived novel transcripts for both Illumina and four quantification methods for PB Kinnex (Methods), along with related paper outputs have been deposited to Zenodo. Other results that either required controlled access deposition or were too large for deposition via Zenodo are available upon demand. All code to reproduce experiments, figures, and tables is available on Github.

## References

[1] Monzó C., Liu T., Conesa A.: Transcriptomics in the era of long-read sequencing. Nature Reviews Genetics, 1–21 (2025) 10.1038/s41576-025-00828-z 40155769

[2] Chen Y., Sim A., Wan Y.K., Yeo K., Lee J.J.X., Ling M.H., Love M.I., Göke J.: Context-aware transcript quantification from long-read rna-seq data with bambu. Nature methods 20(8), 1187–1195 (2023) 37308696 10.1038/s41592-023-01908-w PMC10448944

[3] Prjibelski A.D., Mikheenko A., Joglekar A., Smetanin A., Jarroux J., Lapidus A.L., Tilgner H.U.: Accurate isoform discovery with isoquant using long reads. Nature Biotechnology 41(7), 915–918 (2023) 10.1038/s41587-022-01565-y PMC10344776 36593406

[4] Dong X., Du M.R., Gouil Q., Tian L., Jabbari J.S., Bowden R., Baldoni P.L., Chen Y., Smyth G.K., Amarasinghe S.L., : Benchmarking long-read rna-sequencing analysis tools using in silico mixtures. Nature Methods 20(11), 1810–1821 (2023) 37783886 10.1038/s41592-023-02026-3

[5] Pardo-Palacios F.J., Wang D., Reese F., Diekhans M., Carbonell-Sala S., Williams B., Loveland J.E., De María M., Adams M.S., Balderrama-Gutierrez G., : Systematic assessment of long-read rna-seq methods for transcript identification and quantification. Nature methods 21(7), 1349–1363 (2024) 38849569 10.1038/s41592-024-02298-3 PMC11543605

[6] Sharon D., Tilgner H., Grubert F., Snyder M.: A single-molecule long-read survey of the human transcriptome. Nat. Biotechnol. 31(11), 1009–1014 (2013) 24108091 10.1038/nbt.2705 PMC4075632

[7] Dijk E.L., Jaszczyszyn Y., Naquin D., Thermes C.: The third revolution in sequencing technology. Trends Genet. 34(9), 666–681 (2018) 29941292 10.1016/j.tig.2018.05.008

[8] Masuda K., Sota Y., Matsuda H.: Gene fusion detection in long-read transcriptome datasets from multiple cancer cell lines. Front. Biosci. (Landmark Ed.) 29(12), 413 (2024) 39735992 10.31083/j.fbl2912413

[9] Sakamoto Y., Miyake S., Oka M., Kanai A., Kawai Y., Nagasawa S., Shiraishi Y., Tokunaga K., Kohno T., Seki M., Suzuki Y., Suzuki A.: Phasing analysis of lung cancer genomes using a long read sequencer. Nat. Commun. 13(1), 3464 (2022) 35710642 10.1038/s41467-022-31133-6 PMC9203510

[10] Su Y., Yu Z., Jin S., Ai Z., Yuan R., Chen X., Xue Z., Guo Y., Chen D., Liang H., : Comprehensive assessment of mrna isoform detection methods for long-read sequencing data. Nature Communications 15(1), 3972 (2024) 10.1038/s41467-024-48117-3 PMC11087464 38730241

[11] Chen Y., Davidson N.M., Wan Y.K., Yao F., Su Y., Gamaarachchi H., Sim A., Patel H., Low H.M., Hendra C., : A systematic benchmark of nanopore long-read rna sequencing for transcript-level analysis in human cell lines. Nature methods, 1–12 (2025) 40082608 10.1038/s41592-025-02623-4 PMC11978509

[12] Al’Khafaji A.M., Smith J.T., Garimella K.V., Babadi M., Popic V., Sade-Feldman M., Gatzen M., Sarkizova S., Schwartz M.A., Blaum E.M., : High-throughput rna isoform sequencing using programmed cdna concatenation. Nature biotechnology 42(4), 582–586 (2024) 10.1038/s41587-023-01815-7 PMC12236355 37291427

[13] Nelson E.A., Qiu J., Chavkin N.W., Hirschi K.K.: Directed differentiation of hemogenic endothelial cells from human pluripotent stem cells. J. Vis. Exp. (169) (2021) 10.3791/62391 PMC8675434 33871448

[14] Zhang C., Li H., Wang S.: Common gene signatures and molecular mechanisms of diabetic nephropathy and metabolic syndrome. Front. Public Health 11, 1150122 (2023) 37143982 10.3389/fpubh.2023.1150122 PMC10151256

[15] Romero M.F., Chen A.-P., Parker M.D., Boron W.F.: The slc4 family of bicarbonate hco transporters. Mol. Aspects Med. 34(2–3), 159–182 (2013) 23506864 10.1016/j.mam.2012.10.008 PMC3605756

[16] Liao Z., Cantor J.M.: Endothelial cells require CD98 for efficient angiogenesis-brief report. Arterioscler. Thromb. Vasc. Biol. 36(11), 2163–2166 (2016) 27687603 10.1161/ATVBAHA.116.308335 PMC5954993

[17] Nichol D., Stuhlmann H.: EGFL7: a unique angiogenic signaling factor in vascular development and disease. Blood 119(6), 1345–1352 (2012) 22160377 10.1182/blood-2011-10-322446 PMC3286203

[18] Yeh Y.-T., Hur S.S., Chang J., Wang K.-C., Chiu J.-J., Li Y.-S., Chien S.: Matrix stiffness regulates endothelial cell proliferation through septin 9. PLoS One 7(10), 46889 (2012) 10.1371/journal.pone.0046889 PMC3485289 23118862

[19] Pardo-Palacios F.J., Arzalluz-Luque A., Kondratova L., Salguero P., Mestre-Tomás J., Amorín R., Estevan-Morió E., Liu T., Nanni A., McIntyre L., : Sqanti3: curation of long-read transcriptomes for accurate identification of known and novel isoforms. Nature methods 21(5), 793–797 (2024) 38509328 10.1038/s41592-024-02229-2 PMC11093726

[20] Varabyou A., Erdogdu B., Salzberg S.L., Pertea M.: Investigating open reading frames in known and novel transcripts using orfanage. Nature computational science 3(8), 700–708 (2023) 38098813 10.1038/s43588-023-00496-1 PMC10718564

[21] Baldoni P.L., Chen Y., Hediyeh-Zadeh S., Liao Y., Dong X., Ritchie M.E., Shi W., Smyth G.K.: Dividing out quantification uncertainty allows efficient assessment of differential transcript expression with edger. Nucleic Acids Research 52(3), 13–13 (2024) 10.1093/nar/gkad1167 PMC10853777 38059347

[22] Amarasinghe S.L., Ritchie M.E., Gouil Q.: long-read-tools. org: an interactive catalogue of analysis methods for long-read sequencing data. GigaScience 10(2), 003 (2021) 10.1093/gigascience/giab003 PMC7931822 33590862

[23] Patro R., Duggal G., Love M.I., Irizarry R.A., Kingsford C.: Salmon provides fast and bias-aware quantification of transcript expression. Nature methods 14(4), 417–419 (2017) 28263959 10.1038/nmeth.4197 PMC5600148

[24] Loving R.K., Sullivan D.K., Booeshagi A.S., Reese F., Rebboah E., Sakr J., Rezaie N., Liang H.Y., Filimban G., Kawauchi S., : Long-read sequencing transcriptome quantification with lr-kallisto. bioRxiv, 2024–07 (2025) 10.1371/journal.pcbi.1013692 PMC12680354 41325434

[25] JousheghaniZ.Z., PatroR.: Oarfish: Enhanced probabilistic modeling leads to improved accuracy in long read transcriptome quantification. bioRxiv (2024)10.1093/bioinformatics/btaf24040662837

[26] Soneson C., Matthes K.L., Nowicka M., Law C.W., Robinson M.D.: Isoform prefiltering improves performance of count-based methods for analysis of differential transcript usage. Genome Biol. 17, 12 (2016) 26813113 10.1186/s13059-015-0862-3 PMC4729156

[27] Trapnell C., Hendrickson D.G., Sauvageau M., Goff L., Rinn J.L., Pachter L.: Differential analysis of gene regulation at transcript resolution with RNA-seq. Nat. Biotechnol. 31(1), 46–53 (2013) 23222703 10.1038/nbt.2450 PMC3869392

[28] Pimentel H., Bray N.L., Puente S., Melsted P., Pachter L.: Differential analysis of rna-seq incorporating quantification uncertainty. Nature methods 14(7), 687–690 (2017) 28581496 10.1038/nmeth.4324

[29] Dong X., Du M.R.M., Gouil Q., Tian L., Jabbari J.S., Bowden R., Baldoni P.L., Chen Y., Smyth G.K., Amarasinghe S.L., Law C.W., Ritchie M.E.: Benchmarking long-read RNA-sequencing analysis tools using in silico mixtures. Nat. Methods 20(11), 1810–1821 (2023) 37783886 10.1038/s41592-023-02026-3

[30] Zhu A., Srivastava A., Ibrahim J.G., Patro R., Love M.I.: Nonparametric expression analysis using inferential replicate counts. Nucleic Acids Research 47(18), 105–105 (2019) 10.1093/nar/gkz622 PMC6765120 31372651

[31] Qiu J., Nordling S., Vasavada H.H., Butcher E.C., Hirschi K.K.: Retinoic acid promotes endothelial cell cycle early G1 state to enable human hemogenic endothelial cell specification. Cell Rep. 33(9), 108465 (2020) 33264627 10.1016/j.celrep.2020.108465 PMC8105879

[32] Dobin A., Davis C.A., Schlesinger F., Drenkow J., Zaleski C., Jha S., Batut P., Chaisson M., Gingeras T.R.: Star: ultrafast universal rna-seq aligner. Bioinformatics 29(1), 15–21 (2013) 23104886 10.1093/bioinformatics/bts635 PMC3530905

[33] Li H., Handsaker B., Wysoker A., Fennell T., Ruan J., Homer N., Marth G., Abecasis G., Durbin R., Subgroup, .G.P.D.P.: The sequence alignment/map format and samtools. bioinformatics 25(16), 2078–2079 (2009) 19505943 10.1093/bioinformatics/btp352 PMC2723002

[34] Li H.: Minimap2: pairwise alignment for nucleotide sequences. Bioinformatics 34(18), 3094–3100 (2018) 29750242 10.1093/bioinformatics/bty191 PMC6137996

[35] Frankish A., Carbonell-Sala S., Diekhans M., Jungreis I., Loveland J.E., Mudge J.M., Sisu C., Wright J.C., Arnan C., Barnes I., : Gencode: reference annotation for the human and mouse genomes in 2023. Nucleic acids research 51(D1), 942–949 (2023) 10.1093/nar/gkac1071 PMC9825462 36420896

[36] Robinson M.D., McCarthy D.J., Smyth G.K.: edger: a bioconductor package for differential expression analysis of digital gene expression data. bioinformatics 26(1), 139–140 (2010) 19910308 10.1093/bioinformatics/btp616 PMC2796818

[37] Wang L., Wang S., Li W.: Rseqc: quality control of rna-seq experiments. Bioinformatics 28(16), 2184–2185 (2012) 22743226 10.1093/bioinformatics/bts356

[38] Pertea M., Pertea G.M., Antonescu C.M., Chang T.-C., Mendell J.T., Salzberg S.L.: Stringtie enables improved reconstruction of a transcriptome from rna-seq reads. Nature biotechnology 33(3), 290–295 (2015) 10.1038/nbt.3122 PMC4643835 25690850

[39] Pertea G., Pertea M.: Gff utilities: Gffread and gffcompare. F1000Research 9, (2020) 10.12688/f1000research.23297.1 PMC7222033 32489650

[40] Ritchie M.E., Phipson B., Wu D., Hu Y., Law C.W., Shi W., Smyth G.K.: limma powers differential expression analyses for rna-sequencing and microarray studies. Nucleic acids research 43(7), 47–47 (2015) 10.1093/nar/gkv007 PMC4402510 25605792

[41] Köster J., Rahmann S.: Snakemake—a scalable bioinformatics workflow engine. Bioinformatics 28(19), 2520–2522 (2012) 22908215 10.1093/bioinformatics/bts480

[42] R Core Team: R: A Language and Environment for Statistical Computing. R Foundation for Statistical Computing, Vienna, Austria (2025). R Foundation for Statistical Computing. https://www.R-project.org/

[43] Wickham H.: Ggplot2: Elegant Graphics for Data Analysis. Springer, ??? (2016). https://ggplot2.tidyverse.org

[44] Steenwyk J.L., Rokas A.: ggpubfigs: colorblind-friendly color palettes and ggplot2 graphic system extensions for publication-quality scientific figures. Microbiology Resource Announcements 10(44), 10–1128 (2021) 10.1128/MRA.00871-21 PMC8567791 34734767

