# Supplementary material for: A Systematic Benchmark of High-Accuracy PacBio Long-Read RNA Sequencing for Transcript-Level Quantification
Source: bioRxiv. 2025 Jun 30:2025.05.30.656561. Originally published 2025 Jun 2. Preprint. [Version 2] doi: 10.1101/2025.05.30.656561 (PMC12157514; doi:10.1101/2025.05.30.656561)
Supplement: 1 [file NIHPP2025.05.30.656561V2-supplement-1.pdf]

bioRxiv preprint doi: https://doi.org/10.1101/2025.05.30.656561; this version posted June 30, 2025. The copyright holder for this preprint (which was not certified by peer review) is the author/funder, who has granted bioRxiv a license to display the preprint in perpetuity. It is made available under aCC-BY 4.0 International license.

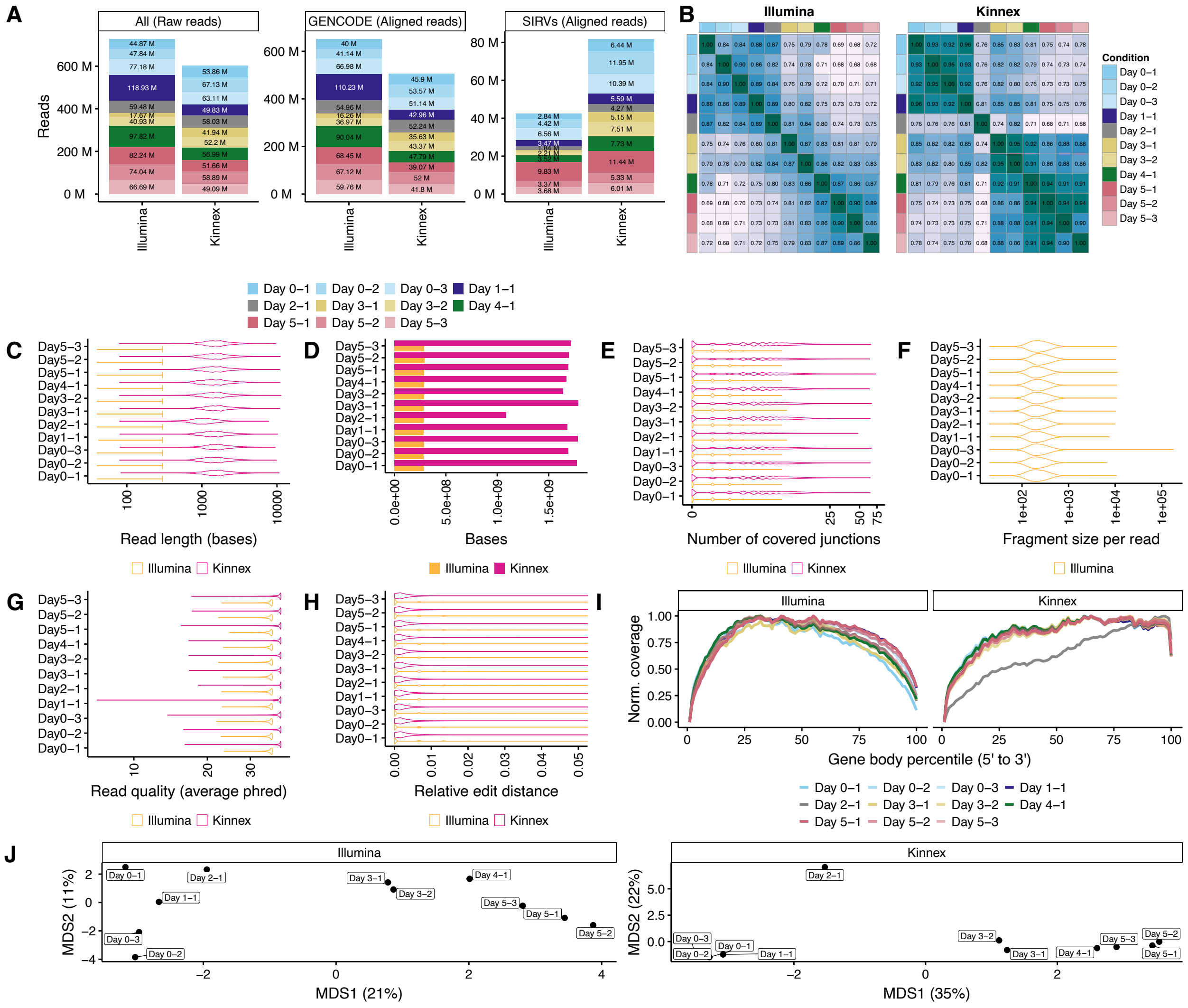

Figure S1: Quality control of all samples in our dataset highlights quality issues in Day2-1 for Kinnex. A. Number of raw and aligned reads per technology, stratified by their source across all samples. Day 3-1 had 0.64M aligned SIRV reads (not shown due to space). **B.** Spearman correlation heatmap of transcript-level quantification across all samples, highlighting sample-similarities across the full differentiation for each technology and quality issues with Day 2-1 for Kinnex. **C.** Read lengths of 1M randomly sampled reads aligning to the human genome, stratified by technology for all samples, highlighting quality issues with Day2-1 for Kinnex. For Illumina, the length was calculated across both ends. **D.** Number of base pairs sequenced from 1M randomly sampled reads collected for each technology (same reads as **C**). **E.** Number of covered junctions per read from 1M randomly sampled reads collected for each technology (same reads as **C**). **F.** Fragment size distribution of 1M randomly sampled reads collected for each technology (same reads as **C**). **G.** Average base quality of 1M randomly sampled reads aligning to the human genome, stratified by technology for all days (same reads as **C**). Average base quality for Illumina was determined from both ends. **H.** Empirical base quality of 1M randomly sampled reads collected for each technology (same reads as **C**). **I.** Normalized coverage of reads aligning to 10,000 randomly sampled GENCODE transcripts across gene body percentiles, stratified by technology, across all samples, highlighting quality issues with Kinnex Day2-1. **J.** Transcript-level quantification-based MDS plots highlighting quality issues for Day2-1 for Kinnex, stratified by technology.

bioRxiv preprint doi: https://doi.org/10.1101/2025.05.30.656561; this version posted June 30, 2025. The copyright holder for this preprint (which was not certified by peer review) is the author/funder, who has granted bioRxiv a license to display the preprint in perpetuity. It is made available under aCC-BY 4.0 International license.

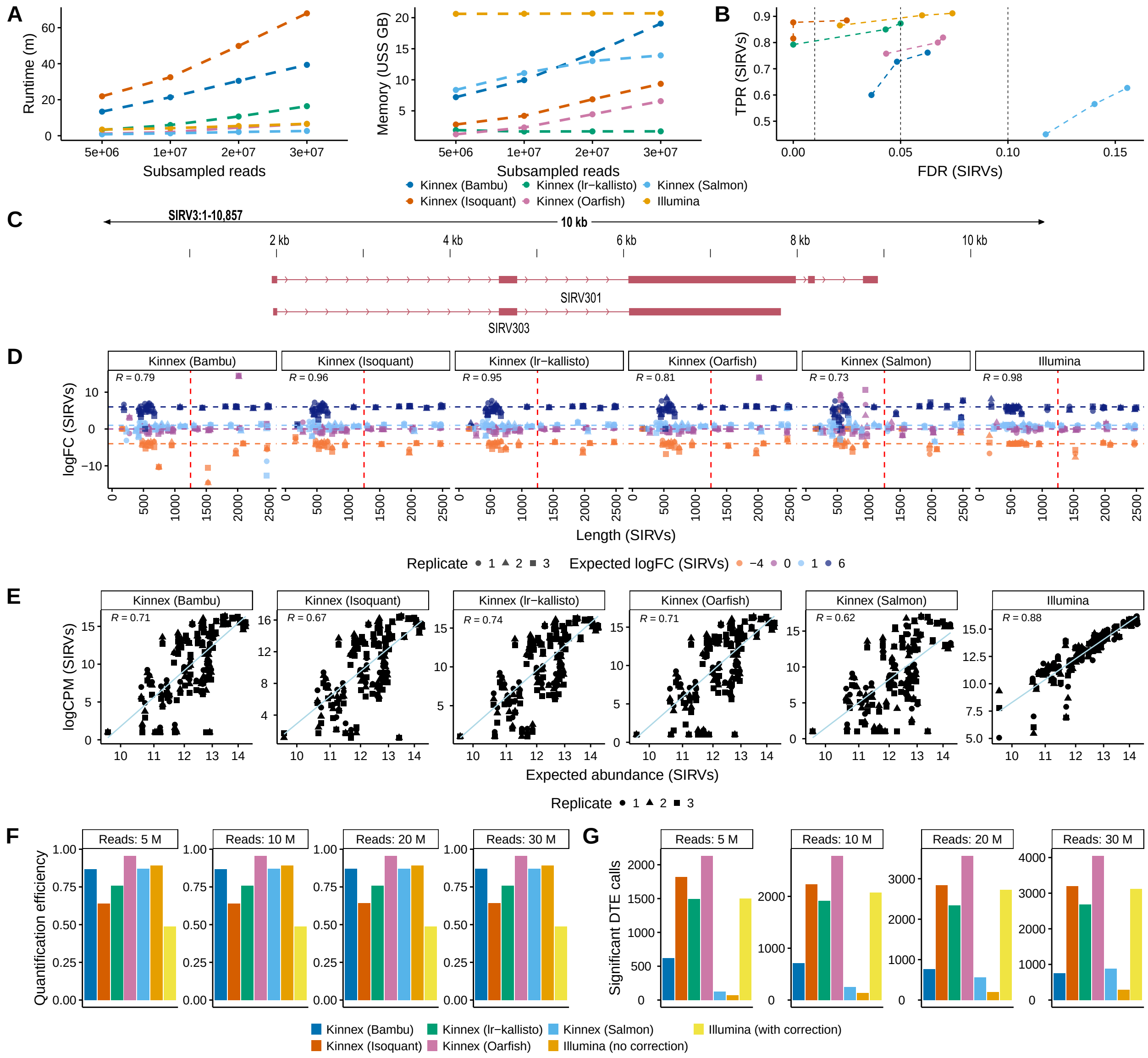

Figure S2: **Comparison between different Kinnex quantification methods. A.** Computational requirements of quantification methods for Kinnex and Illumina in terms of runtime (left) and memory (right) across all days and replicates of differentiation after downsampling the GENCODE-aligned data of each day to a fixed read depth (see **Methods**). Alignment and index creation were not included within computational requirements. **B.** True positive rates (TPRs) and false discovery rates (FDRs) for Kinnex and Illumina quantification methods on Differential Transcript Expression (DTE) between Day 0 (E1 mix) and Day 5 (E2 mix) on the SIRV spike-ins when downsampling to a fixed depth of 1M reads for all samples (see **Methods**). **C.** Browser track highlighting an example of a pair of problematic SIRV transcripts that may cause outliers for some Kinnex lrRNA-seq quantification methods. **D.** Relative quantification results between the same replicate on Day 0 (E1 mix) versus Day 5 (E2 mix) of four quantification methods for Kinnex lrRNA-seq and Illumina as measured by similarity of observed and expected log-fold changes between SIRV spike-in mixes across SIRV transcript length when downsampling to a fixed depth of 1M reads for all samples (see **Methods**). **E.** Absolute quantification of four quantification methods for Kinnex lrRNA-seq and Illumina as measured by concordance of observed and expected absolute abundances for each replicate on Day 0 (E1 mix) when downsampling to a fixed depth of 1M reads for all samples (see **Methods**). R denotes Pearson correlation. **F.** Relative quantification efficiency, computed as number of counts divided by number of raw reads, of four quantification methods for Kinnex lrRNA-seq and Illumina, stratified by the downsampled number of input reads. **G.** Number of significant DTE calls that overlap with at least one other method at the same depth of four quantification methods for Kinnex lrRNA-seq and Illumina, stratified by the downsampled number of input reads. R denotes Pearson correlation.

bioRxiv preprint doi: https://doi.org/10.1101/2025.05.30.656561; this version posted June 30, 2025. The copyright holder for this preprint (which was not certified by peer review) is the author/funder, who has granted bioRxiv a license to display the preprint in perpetuity. It is made available under aCC-BY 4.0 International license.

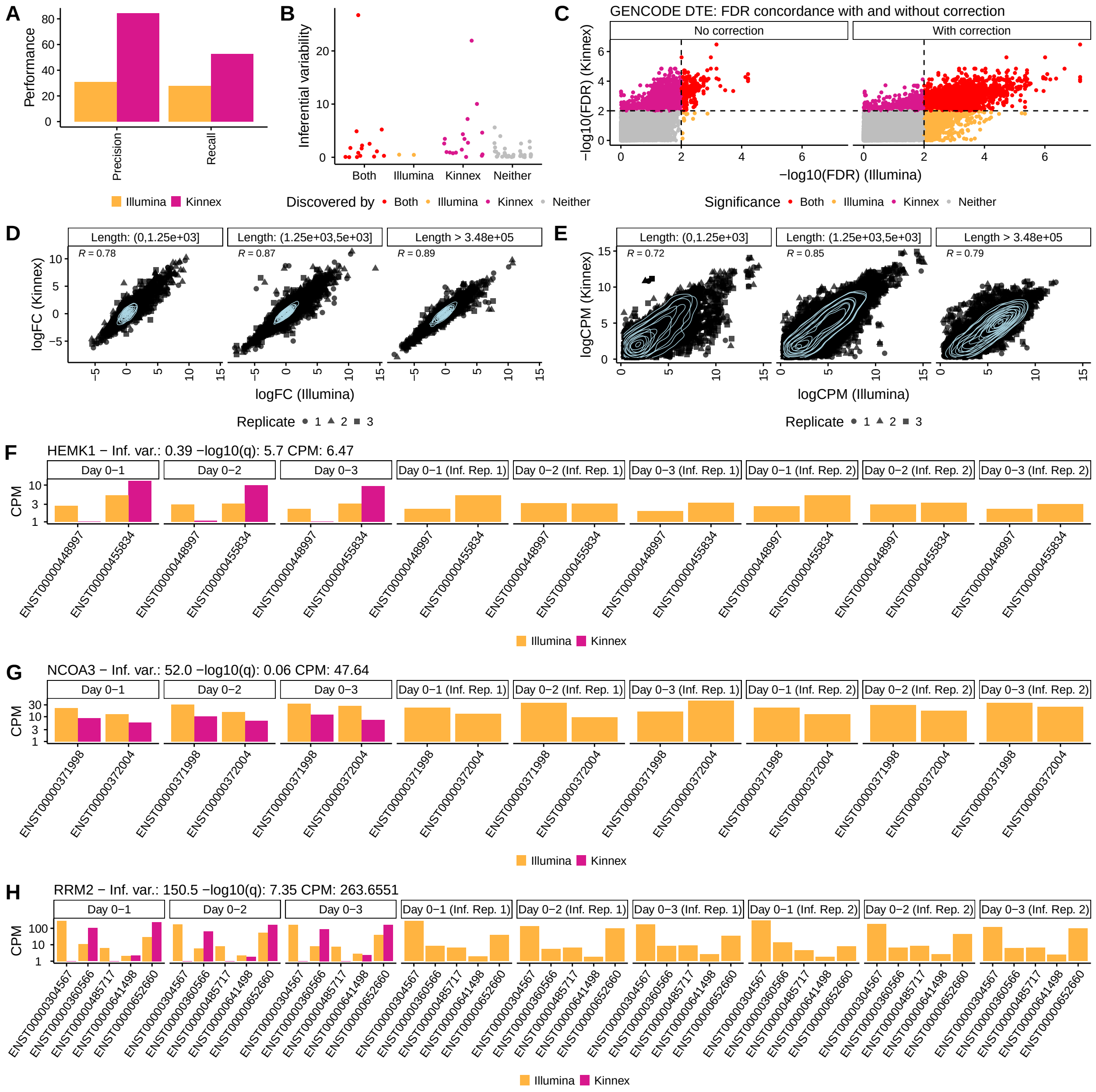

Figure S3: **Performance of Kinnex relative to Illumina on transcript discovery, DTE, and relative and absolute quantification. A.** Precision and recall of de-novo transcript discovery on three Day 0 (E1 mix) SIRV replicates by technology. Both platforms were ran on downsampled read sets consisting of 2.5 M reads. **B.** Mean inferential variability of SIRV transcripts on three Day 0 replicates by whether they were discovered by both platforms, only Illumina, only Kinnex, or neither platform. **C.** Concordance in per-transcript DTE q-values between Illumina and Kinnex with and without applying an inferential variability correction for Illumina [16] (see **Methods**). Only transcripts that were not filtered for all technologies (Kinnex, Illumina without correction, Illumina with correction) are shown. **D.** Concordance of per-replicate per-transcript log-fold changes between Kinnex and Illumina with length in nts. **E.** Concordance of per-replicate per-transcript log CPM values between Kinnex and Illumina. For fairness, in panels C-E, both technologies were downsampled to 30M reads. **F.** Exemplary quantification of the three Day 0 replicates for Illumina and Kinnex and two inferential Day 0 replicates for Illumina on the *HEMK1* gene. **G.** Exemplary quantification of the three Day 0 replicates for Illumina and Kinnex and two inferential Day 0 replicates for Illumina on the *NCOA3* gene. **H.** Exemplary quantification of the three Day 0 replicates for Illumina and Kinnex and two inferential Day 0 replicates for Illumina on the *RRM2* gene. R denotes Pearson correlation.
